# *tert*-Butyl 2-(4-nitro­phen­oxy)acetate

**DOI:** 10.1107/S1600536811003229

**Published:** 2011-01-29

**Authors:** Qamar Ali, Itrat Anis, M. Raza Shah, Seik Weng Ng

**Affiliations:** a H.E.J. Research Institute of Chemistry, International Center for Chemical and Biological Sciences, University of Karachi, Karachi 7527, Pakistan; b Department of Chemistry, University of Malaya, 50603 Kuala Lumpur, Malaysia

## Abstract

In the title mol­ecule, C_12_H_15_NO_5_, the nitro­phen­oxy portion is approximately planar (r.m.s. deviation = 0.034 Å) and makes an angle of 84.8 (1)° with respect to the –CH_2_–C(=O)–O–C fragment. In the crystal, π–π stacking is observed between nearly parallel benzene rings of adjacent mol­ecules, the centroid–centroid distance being 3.6806 (10) Å. Weak inter­molecular C—H⋯O hydrogen bonding is present in the crystal structure.

## Related literature

For a study of the biopotency of the title compound, see: Arfan *et al.* (2010 ▶). For related structures, see: Ali *et al.* (2010 ▶); Mustafa *et al.* (2009 ▶).
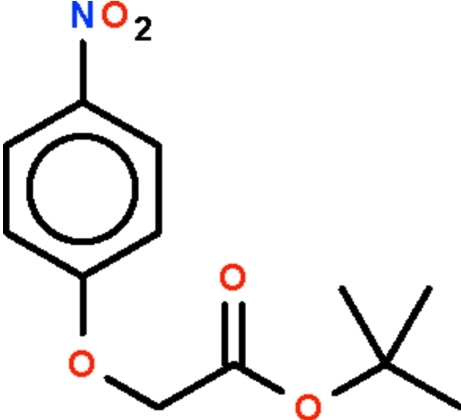


## Experimental

### 

#### Crystal data

C_12_H_15_NO_5_
*M* *_r_* = 253.25
Monoclinic, 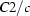

*a* = 19.2761 (7) Å
*b* = 12.1131 (4) Å
*c* = 11.7267 (5) Å
β = 111.682 (4)°
*V* = 2544.38 (17) Å^3^
*Z* = 8
Mo *K*α radiation
μ = 0.10 mm^−1^
*T* = 100 K
0.30 × 0.20 × 0.05 mm

#### Data collection

Agilent SuperNova Dual diffractometer with an Atlas detector
Absorption correction: multi-scan (*CrysAlis PRO*; Agilent, 2010 ▶) *T*_min_ = 0.664, *T*_max_ = 1.000
5580 measured reflections
2824 independent reflections
2075 reflections with *I* > 2σ(*I*)
*R*_int_ = 0.029

#### Refinement

*R*[*F* ^2^ > 2σ(*F* ^2^)] = 0.047
*wR*(*F* ^2^) = 0.120
*S* = 1.06
2824 reflections
164 parameters
H-atom parameters constrained
Δρ_max_ = 0.27 e Å^−3^
Δρ_min_ = −0.21 e Å^−3^

### 

Data collection: *CrysAlis PRO* (Agilent, 2010 ▶); cell refinement: *CrysAlis PRO*; data reduction: *CrysAlis PRO*; program(s) used to solve structure: *SHELXS97* (Sheldrick, 2008 ▶); program(s) used to refine structure: *SHELXL97* (Sheldrick, 2008 ▶); molecular graphics: *X-SEED* (Barbour, 2001 ▶); software used to prepare material for publication: *publCIF* (Westrip, 2010 ▶).

## Supplementary Material

Crystal structure: contains datablocks global, I. DOI: 10.1107/S1600536811003229/xu5151sup1.cif

Structure factors: contains datablocks I. DOI: 10.1107/S1600536811003229/xu5151Isup2.hkl

Additional supplementary materials:  crystallographic information; 3D view; checkCIF report

## Figures and Tables

**Table 1:** Hydrogen-bond geometry (Å, °)

*D*—H⋯*A*	*D*—H	H⋯*A*	*D*⋯*A*	*D*—H⋯*A*
C6—H6⋯O4^i^	0.95	2.50	3.201 (2)	130
C12—H12*C*⋯O2^ii^	0.98	2.55	3.489 (3)	161

Symmetry codes: (i) 
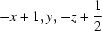
; (ii) 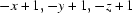.

**Fig. 1.**
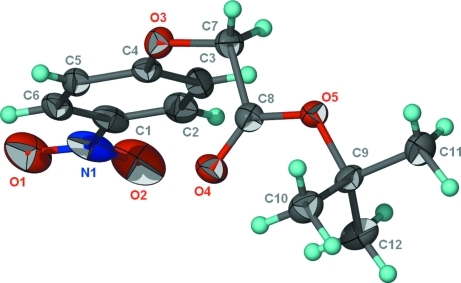
Thermal ellipsoid plot (Barbour, 2001) of at C12H15NO5 the 70% probability level; hydrogen atoms are drawn as spheres of arbitrary radius.

## supplementary crystallographic information

### Comment

The C_12_H_15_NO_5_ compound (Scheme I) was synthesized for evaluation of its potency against urease enzymes (Arfan *et al.*, 2010); we have also synthesized other *t*-butyl esters of phenols (Ali *et al.*, 2010; Mustafa *et al.*, 2010). The nitrophenoxy portion is approximately planar (r.m.s. deviation 0.034 Å) this makes an angle of 84.8 (1)° with respect to the –CH_2_–C(═O)–O–C fragment (Fig. 1). π-π stacking is observed between nearly parallel C1-benzene and C1^i^-benzene rings of adjacent molecules (symmetry code: (i) 1-x, y, 1/2-z), centroids distance being 3.6806 (10) Å. Intermolecular weak C—H···O hydrogen bonding is present in the crystal structure (Table 1).

### Experimental

4-Nitrophenol (1 g, 7 mmol) was dissolved in acetone (25 ml). To the solution was added potassium carbonate (2 g, 14 mmol). *t*-Butyl bromoacetate (2 ml, 14 mmol) was added amd the mixture heated for 3 hurs. The solvent was evaporated and the residue was dissolved in a mixture of water (50 ml) and ethyl acetate (50 ml). The aqueous layer was extracted three times with ethyl acetate. The combined organic phases were evaporated under reduced pressure and the solid material was recrystallized from *n*-hexane to give the produce in 80% yield.

### Refinement

Carbon-bound H-atoms were placed in calculated positions [C—H 0.95 to 0.98 Å, *U*_iso_(H) 1.2 to 1.5*U*_eq_(C)] and were included in the refinement in the riding model approximation.

### Crystal data

**Table:**

C_12_H_15_NO_5_	*F*(000) = 1072
*M**_r_* = 253.25	*D*_x_ = 1.322 Mg m^−^^3^
Monoclinic, *C*2/*c*	Mo *K*α radiation, λ = 0.71073 Å
Hall symbol: -C 2yc	Cell parameters from 1942 reflections
*a* = 19.2761 (7) Å	θ = 2.3–28.5°
*b* = 12.1131 (4) Å	µ = 0.10 mm^−^^1^
*c* = 11.7267 (5) Å	*T* = 100 K
β = 111.682 (4)°	Plate, colorless
*V* = 2544.38 (17) Å^3^	0.30 × 0.20 × 0.05 mm
*Z* = 8	

### Data collection

**Table:**

Agilent SuperNova Dual diffractometer with an Atlas detector	2824 independent reflections
Radiation source: SuperNova (Mo) X-ray Source	2075 reflections with *I* > 2σ(*I*)
Mirror	*R*_int_ = 0.029
Detector resolution: 10.4041 pixels mm^-1^	θ_max_ = 27.5°, θ_min_ = 2.3°
ω scans	*h* = −17→24
Absorption correction: multi-scan (*CrysAlis PRO*; Agilent, 2010)	*k* = −13→15
*T*_min_ = 0.664, *T*_max_ = 1.000	*l* = −14→14
5580 measured reflections	

### Refinement

**Table:**

Refinement on *F*^2^	Secondary atom site location: difference Fourier map
Least-squares matrix: full	Hydrogen site location: inferred from neighbouring sites
*R*[*F*^2^ > 2σ(*F*^2^)] = 0.047	H-atom parameters constrained
*wR*(*F*^2^) = 0.120	*w* = 1/[σ^2^(*F*_o_^2^) + (0.0444*P*)^2^ + 0.6948*P*] where *P* = (*F*_o_^2^ + 2*F*_c_^2^)/3
*S* = 1.06	(Δ/σ)_max_ = 0.001
2824 reflections	Δρ_max_ = 0.27 e Å^−^^3^
164 parameters	Δρ_min_ = −0.21 e Å^−^^3^
0 restraints	Extinction correction: *SHELXL97* (Sheldrick, 2008), Fc^*^=kFc[1+0.001xFc^2^λ^3^/sin(2θ)]^-1/4^
Primary atom site location: structure-invariant direct methods	Extinction coefficient: 0.0033 (4)

### Fractional atomic coordinates and isotropic or equivalent isotropic displacement parameters (Å^2^)

**Table:**

	*x*	*y*	*z*	*U*_iso_*/*U*_eq_	
O1	0.37259 (7)	0.48179 (13)	0.04938 (14)	0.0505 (4)	
O2	0.41306 (9)	0.58588 (13)	0.21067 (16)	0.0581 (5)	
O3	0.44798 (6)	0.11669 (9)	0.44903 (11)	0.0251 (3)	
O4	0.59965 (6)	0.13203 (9)	0.53127 (11)	0.0279 (3)	
O5	0.60685 (5)	0.11686 (9)	0.72849 (10)	0.0233 (3)	
N1	0.39810 (8)	0.49483 (14)	0.16138 (17)	0.0387 (4)	
C1	0.41151 (9)	0.39685 (14)	0.23939 (17)	0.0275 (4)	
C2	0.43579 (9)	0.41028 (15)	0.36502 (17)	0.0287 (4)	
H2	0.4428	0.4821	0.4001	0.034*	
C3	0.44980 (8)	0.31747 (14)	0.43918 (16)	0.0258 (4)	
H3	0.4666	0.3249	0.5258	0.031*	
C4	0.43901 (8)	0.21342 (13)	0.38562 (15)	0.0221 (4)	
C5	0.41454 (8)	0.20196 (14)	0.25872 (15)	0.0240 (4)	
H5	0.4076	0.1304	0.2231	0.029*	
C6	0.40040 (8)	0.29378 (15)	0.18461 (16)	0.0275 (4)	
H6	0.3834	0.2866	0.0979	0.033*	
C7	0.48824 (8)	0.11660 (14)	0.57817 (15)	0.0250 (4)	
H7A	0.4768	0.0482	0.6141	0.030*	
H7B	0.4723	0.1803	0.6155	0.030*	
C8	0.57178 (8)	0.12344 (13)	0.60739 (16)	0.0223 (4)	
C9	0.69011 (8)	0.12075 (13)	0.78688 (16)	0.0241 (4)	
C10	0.72379 (9)	0.02283 (15)	0.74544 (17)	0.0312 (4)	
H10A	0.7142	0.0297	0.6576	0.047*	
H10B	0.7012	−0.0455	0.7602	0.047*	
H10C	0.7778	0.0210	0.7917	0.047*	
C11	0.70230 (9)	0.11251 (16)	0.92190 (16)	0.0352 (5)	
H11A	0.6833	0.0415	0.9379	0.053*	
H11B	0.6757	0.1727	0.9440	0.053*	
H11C	0.7558	0.1180	0.9711	0.053*	
C12	0.71824 (9)	0.23046 (15)	0.75805 (18)	0.0341 (5)	
H12A	0.7088	0.2344	0.6701	0.051*	
H12B	0.7720	0.2367	0.8049	0.051*	
H12C	0.6921	0.2910	0.7806	0.051*	

### Atomic displacement parameters (Å^2^)

**Table:**

	*U*^11^	*U*^22^	*U*^33^	*U*^12^	*U*^13^	*U*^23^
O1	0.0487 (8)	0.0646 (11)	0.0413 (10)	0.0150 (7)	0.0201 (7)	0.0271 (8)
O2	0.0813 (11)	0.0317 (9)	0.0778 (13)	0.0110 (7)	0.0485 (10)	0.0148 (9)
O3	0.0243 (6)	0.0262 (7)	0.0213 (6)	−0.0002 (4)	0.0044 (5)	0.0025 (5)
O4	0.0270 (6)	0.0348 (7)	0.0239 (7)	−0.0001 (5)	0.0118 (5)	0.0043 (6)
O5	0.0185 (6)	0.0303 (7)	0.0201 (6)	−0.0014 (4)	0.0059 (5)	0.0008 (5)
N1	0.0367 (9)	0.0391 (10)	0.0513 (12)	0.0145 (7)	0.0290 (8)	0.0197 (9)
C1	0.0242 (8)	0.0314 (10)	0.0315 (10)	0.0087 (7)	0.0159 (7)	0.0098 (8)
C2	0.0289 (9)	0.0263 (9)	0.0348 (11)	0.0030 (7)	0.0162 (8)	−0.0010 (8)
C3	0.0269 (8)	0.0295 (10)	0.0220 (9)	0.0017 (7)	0.0100 (7)	−0.0013 (8)
C4	0.0165 (7)	0.0269 (9)	0.0233 (9)	0.0020 (6)	0.0078 (6)	0.0037 (7)
C5	0.0196 (8)	0.0305 (9)	0.0221 (9)	0.0020 (6)	0.0079 (6)	−0.0023 (7)
C6	0.0189 (8)	0.0424 (11)	0.0226 (9)	0.0052 (7)	0.0093 (7)	0.0036 (8)
C7	0.0224 (8)	0.0321 (10)	0.0188 (9)	−0.0002 (6)	0.0057 (7)	0.0043 (8)
C8	0.0237 (8)	0.0203 (8)	0.0223 (9)	−0.0002 (6)	0.0078 (7)	0.0019 (7)
C9	0.0171 (8)	0.0275 (9)	0.0247 (9)	−0.0023 (6)	0.0043 (7)	−0.0019 (7)
C10	0.0232 (8)	0.0327 (10)	0.0357 (11)	0.0011 (7)	0.0086 (7)	−0.0032 (9)
C11	0.0242 (9)	0.0523 (13)	0.0250 (10)	−0.0014 (8)	0.0044 (7)	−0.0021 (9)
C12	0.0261 (9)	0.0323 (10)	0.0422 (12)	−0.0054 (7)	0.0105 (8)	−0.0013 (9)

### Geometric parameters (Å, °)

**Table:**

O1—N1	1.231 (2)	C6—H6	0.9500
O2—N1	1.229 (2)	C7—C8	1.520 (2)
O3—C4	1.3641 (19)	C7—H7A	0.9900
O3—C7	1.424 (2)	C7—H7B	0.9900
O4—C8	1.2044 (19)	C9—C10	1.516 (2)
O5—C8	1.3307 (19)	C9—C11	1.516 (2)
O5—C9	1.4949 (18)	C9—C12	1.520 (2)
N1—C1	1.462 (2)	C10—H10A	0.9800
C1—C2	1.381 (3)	C10—H10B	0.9800
C1—C6	1.384 (2)	C10—H10C	0.9800
C2—C3	1.386 (2)	C11—H11A	0.9800
C2—H2	0.9500	C11—H11B	0.9800
C3—C4	1.389 (2)	C11—H11C	0.9800
C3—H3	0.9500	C12—H12A	0.9800
C4—C5	1.392 (2)	C12—H12B	0.9800
C5—C6	1.375 (2)	C12—H12C	0.9800
C5—H5	0.9500		
			
C4—O3—C7	119.34 (12)	H7A—C7—H7B	108.1
C8—O5—C9	121.54 (12)	O4—C8—O5	127.32 (14)
O2—N1—O1	123.29 (17)	O4—C8—C7	124.28 (15)
O2—N1—C1	118.53 (18)	O5—C8—C7	108.39 (14)
O1—N1—C1	118.17 (18)	O5—C9—C10	110.06 (12)
C2—C1—C6	122.33 (16)	O5—C9—C11	101.67 (12)
C2—C1—N1	118.94 (17)	C10—C9—C11	111.34 (15)
C6—C1—N1	118.72 (17)	O5—C9—C12	109.67 (13)
C1—C2—C3	119.00 (17)	C10—C9—C12	112.49 (14)
C1—C2—H2	120.5	C11—C9—C12	111.08 (15)
C3—C2—H2	120.5	C9—C10—H10A	109.5
C2—C3—C4	119.37 (16)	C9—C10—H10B	109.5
C2—C3—H3	120.3	H10A—C10—H10B	109.5
C4—C3—H3	120.3	C9—C10—H10C	109.5
O3—C4—C3	124.42 (15)	H10A—C10—H10C	109.5
O3—C4—C5	114.94 (14)	H10B—C10—H10C	109.5
C3—C4—C5	120.59 (15)	C9—C11—H11A	109.5
C6—C5—C4	120.30 (16)	C9—C11—H11B	109.5
C6—C5—H5	119.8	H11A—C11—H11B	109.5
C4—C5—H5	119.8	C9—C11—H11C	109.5
C5—C6—C1	118.40 (16)	H11A—C11—H11C	109.5
C5—C6—H6	120.8	H11B—C11—H11C	109.5
C1—C6—H6	120.8	C9—C12—H12A	109.5
O3—C7—C8	110.82 (14)	C9—C12—H12B	109.5
O3—C7—H7A	109.5	H12A—C12—H12B	109.5
C8—C7—H7A	109.5	C9—C12—H12C	109.5
O3—C7—H7B	109.5	H12A—C12—H12C	109.5
C8—C7—H7B	109.5	H12B—C12—H12C	109.5
			
O2—N1—C1—C2	4.2 (2)	C3—C4—C5—C6	−0.3 (2)
O1—N1—C1—C2	−176.09 (15)	C4—C5—C6—C1	0.4 (2)
O2—N1—C1—C6	−174.92 (15)	C2—C1—C6—C5	−0.3 (2)
O1—N1—C1—C6	4.8 (2)	N1—C1—C6—C5	178.73 (13)
C6—C1—C2—C3	0.2 (2)	C4—O3—C7—C8	−76.68 (17)
N1—C1—C2—C3	−178.85 (13)	C9—O5—C8—O4	0.6 (2)
C1—C2—C3—C4	−0.1 (2)	C9—O5—C8—C7	179.79 (12)
C7—O3—C4—C3	−16.7 (2)	O3—C7—C8—O4	2.6 (2)
C7—O3—C4—C5	165.91 (13)	O3—C7—C8—O5	−176.57 (12)
C2—C3—C4—O3	−177.12 (14)	C8—O5—C9—C10	−62.81 (18)
C2—C3—C4—C5	0.1 (2)	C8—O5—C9—C11	179.09 (14)
O3—C4—C5—C6	177.24 (12)	C8—O5—C9—C12	61.45 (18)

### Hydrogen-bond geometry (Å, °)

**Table:**

*D*—H···*A*	*D*—H	H···*A*	*D*···*A*	*D*—H···*A*
C6—H6···O4^i^	0.95	2.50	3.201 (2)	130
C12—H12C···O2^ii^	0.98	2.55	3.489 (3)	161

Symmetry codes: (i) −*x*+1, *y*, −*z*+1/2; (ii) −*x*+1, −*y*+1, −*z*+1.
